# Implementing electronic health records on a medical service trip improves the patient care process

**DOI:** 10.3389/frhs.2022.960427

**Published:** 2022-09-07

**Authors:** Harm Maarsingh, Kayla Oyler, Gamukama Tuhaise, Mariette Sourial, Adwoa O. Nornoo, Wambazu Moses, Laura A. Rhodes

**Affiliations:** ^1^Department of Pharmaceutical Sciences, Lloyd L. Gregory School of Pharmacy, Palm Beach Atlantic University, West Palm Beach, FL, United States; ^2^Department of Pharmacy Practice, Lloyd L. Gregory School of Pharmacy, Palm Beach Atlantic University, West Palm Beach, FL, United States; ^3^Department of Surgery, Kabale Regional Referral Hospital, Kabale, Uganda; ^4^Kabwohe Clinical Research Centre, Bushenyi, Uganda

**Keywords:** electronic medical records (EMR) systems, patient care process, short-term experiences in global health, medical camp, medical scribe, short-term medical missions, pharmacy

## Abstract

**Background:**

The efficiency of the patient care process of short-term medical service trips is often not assessed. The Gregory School of Pharmacy has organized annual medical camps in rural Uganda, however, the paper health records used for documentation and communication between stations have shown several limitations that hinder an optimal patient care process. Therefore, our objective was to implement an electronic health record system in these medical camps to improve the workflow and optimize the patient care process.

**Methods:**

An electronic health record system that functioned over a battery-operated local area network was developed and implemented. Patient health information was entered and reviewed at the different stations using mobile devices. The impact of electronic health records (used in 2019) on the patient care process was assessed using the number of patients served per physician per hour and the number of prescriptions filled per hour and comparing these to paper records (used in 2017).

**Results:**

Electronic health records were successfully implemented and communication across stations was fluid, thus improving transitions. Importantly, 45% more patients were served per physician per hour and 38% more prescriptions were dispensed per hour when using electronic (2019) compared to paper records (2017), despite having a smaller team in 2019.

**Conclusion:**

Implementation of electronic health records in rural Uganda improved the patient care process and the efficiency of the medical camp.

## Introduction

Providing health care and health services to vulnerable populations around the world through short-term medical missions (STMM) is increasingly popular (1). Annually, around 6,000 STMMs to developing countries are organized from the United States alone (2). In addition to providing medical care, these initiatives may also have social, economic, and diplomatic effects (3), however, the actual impact is rarely assessed–or at least not reported. Indeed, only 6% of the publications regarding medial service trips report data collection, with the outcomes of the reported interventions being often unclear (4). This and the lack of oversight by governmental agencies and accreditation bodies have led to fears regarding unintended harm to the vulnerable people served due to substandard care (4, 5).

STMMs have become part of educational programs in medicine (1), nursing (6), and pharmacy (7, 8) in the form of short-term experiences in global health (9) and offer a great opportunity to participating students to demonstrate their ability to work in an interprofessional setting (10). These service-learning trips do not only impact the people served, but also the participants (11, 12). Students gain awareness of cultural differences, learn to serve vulnerable populations, and gain experience in collaborating in an interprofessional patient care team, which helps them to understand their specific role in the patient care process and to improve their communication skills with other health care practitioners. Despite good intentions, STMMs may be focused more on student learning than on determining beneficial outcomes to the community served (9) and may cause unintentional harm due to lack of follow up, misuse of medications, and potential disenfranchisement of the local health care system (13, 14).

STMMs are an important experiential component of the Lloyd L. Gregory School of Pharmacy, Palm Beach Atlantic University, serving people in Central and South America, Africa, Asia and domestically in South Florida, Georgia and Alaska (7, 15). Since 2013, the School of Pharmacy has provided medical services to people in rural villages in the Mukono district of Uganda, Africa, annually by organizing medical camps operated by a team of health care workers working in different stations (7). Paper health records have been used to document patient information, to communicate between stations, and to serve as the prescription for medications (16). There are several challenges with the paper system that may impact the workflow and the patient care process: transfer of forms and communication between stations (e.g., when diagnoses or medication orders require clarification), the limited space available to write patient information (e.g., when families have multiple dependents or patients have multiple disease states), poor legibility and messiness because of edits, dust or rain.

We anticipated that using electronic health records (EHRs) would resolve these challenges encountered with use of paper health records, thereby improving the flow and communication between the stations and optimizing the patient care process. The use of EHRs and hand-held devices on STMMs has previously been described with most systems being web-based requiring internet access (17). However, internet access in remote places may be unavailable, unreliable, or costly and the impact of EHRs on the patient care process in temporary medical camps in STMMs has not yet been described.

The aim of this study was to investigate the impact of implementing an EHR system that could operate independently without requiring access to electricity or internet on the workflow and the patient care process at medical camps in rural Uganda.

## Materials and methods

### Medical camps

This study was conducted at a series of 1-day medical camps in five rural villages within the Mukono district of Uganda, which the Gregory School of Pharmacy has organized annually since 2013 in collaboration with Ugandan health care workers, the non-profit organization Word In Deed Ministries–that has a continuous local presence promoting education, health, empowerment and entrepreneurship–and local Churches. Most of these rural villages do not have electricity. For the planning and executing of the medical camps, Ugandan physicians and School of Pharmacy faculty communicated regarding the therapeutic drug formulary and the Ugandan guidelines and protocols for common encountered disease states. This approach is in line with the recommendation that STMM teams work together with partners in the host country (18) and that local requirements are met (19).

Faculty (PharmD, PhD, and PharmD residents), PharmD students and alumni (PharmD) from the School of Pharmacy formed an interprofessional healthcare team with Ugandan physicians, nurses, community health workers and interpreters. As part of their training, PharmD students were involved in all steps of the patient care process while being supervised by Faculty and licensed (local) health care workers. Thus, students participated in patient intake, assisted physicians during anamnesis and diagnosing, offered therapeutic recommendations, performed and interpreted point-of-care tests, and filled prescriptions along with counseling on proper medication use. Non-PharmD faculty members were not involved in clinical decision making and were mainly responsible for the logistics, planning, and budget of the STMM as well as overseeing non-clinical activities, such as community events. Dental services and public health services related to HIV, women's health, and family planning were provided by Ugandan agencies and community partners.

Each day, all necessary equipment, devices and medications for the medical camp were set up by the team upon arrival in each village and packed up at the end of the day. The different stations (see Table 1) were typically set up in separate rooms or buildings to provide privacy when meeting the physician and to ensure that unauthorized people do not have access to the medications in the Pharmacy. At arrival, a number was given to each individual patient (when alone) or family unit and patients moved through the camp in order of their number with urgent cases being expedited. Patients moved from the triage station to the physician's station for diagnosis and to receive a prescription order, and finally to the pharmacy to receive their medications and appropriate medication counseling. Because of the separate triage station with data entry, the time the physicians spent with the patients could be maximized. Physicians could send a patient for point-of-care testing (blood glucose levels, malaria, pregnancy status, typhoid, hepatitis B, H. pylori, HIV and syphilis), a process increasingly adopted by medical service trips (20, 21). When verifying pharmacists would observe a potential medication related problem–such as indication without medication, need for optimization of therapy (adjustments in selection, dose, frequency, or duration of therapy), and formulary changes–they would contact the physician to resolve these and the patient would be sent back to the physician for re-evaluation if needed. The Ugandan physicians were the final authority on all clinical decisions.

**Table 1:** Overview and function of the different stations and the different team members involved in the medical camps.

**Station**	**Function**	**Team members**
Waiting area	• Numbers are distributed to each patient (or family). • Public health talks on hygiene and malaria prevention.	Students and interpreters
Intake and triage	Patient information is documented: •Name, age, gender; •PMH, (current) medications, allergies; •Smoking status, alcohol use; •Pregnancy status; •Chief complaint; •Vitals: blood pressure, weight, temperature; •Prayer requests.	Faculty*, students and interpreters
Physician's station	Patients are diagnosed by the physician and the proper medical treatment is selected in consultation with the PharmD (student). If needed, the physician can request blood work for a proper diagnosis or treatment plan.	Ugandan physicians, faculty* and students
Diagnostics lab	Diagnostic tests (blood glucose, pregnancy, malaria, HIV, *H. pylori*, typhoid, hepatitis B and syphilis) requested by the physicians are performed.	Ugandan nurses, faculty* and students
Pharmacy	Checking, filling and verification of the prescriptions to ensure that the proper medication is dispensed. Patients are counseled with respect to proper use of the medication.	Faculty* and students with interpreters

^*^Including PGY1 residents and alumni. PMH, past medical history.

### Paper vs. electronic health records

When using paper records, name(s) and patient number were recorded on the form at patient intake. The form was in letter size format and the front was reserved for the primary family member with room for 3 additional patients on the back (typically dependents such as children). If additional forms were needed to document information, these would be stapled together. Patients brought their forms to each station where pertinent information was added and evaluated. This paper form also served as the prescription in the pharmacy where they were collected at the end of the patient care process. At the conclusion of the STMM, these forms were transported back to the US for data analysis.

The EHRs and a wireless communication network that can be utilized in places that have no electricity or internet access were developed by Infotech Solutions in 2018 using an open-source web platform (TikiWiki), an open-source database platform (MySQL), and an open-source web server (Apache). The EHR structure was designed by members of the mission team using the paper records as a blueprint. When using electronic records, name(s) and patient number were entered at the triage station and the form was sent from station to station digitally over a local area network (LAN) connection. At each station, pertinent patient information was entered and evaluated by accessing the electronic queue where the patient record was residing. After processing the patient information, the record was put in a queue for the next station with a priority queue for urgent cases. In the pharmacy, there were separate queues for filling, verification, and medication counseling. This workflow enabled the team to check patient transitions between stations in real time. Information from up to 10 family members could be documented per record and digital files, such as pictures, could be attached.

The EHR program was installed on a laptop computer (Toshiba Satellite S55–A5294, Minato, Tokyo, Japan). A battery-operated LAN was created with portable routers, signal extenders (TP-Link N300 Wireless Wi-Fi Nano Travel Router, Shenzhen, Guangdong, China) and the laptop as the LAN server. The system allowed multiple devices to use the system simultaneously (we connected up to 12 devices). Tablet computers [iPad WIFI 32GB (5th generation), Apple Inc, Cupertino, CA, USA] with bluetooth keyboards and laptop computers connected to the LAN were used for data entry and evaluation. To ensure patient confidentiality, data were encrypted and the server and electronic devices were password protected. Upon arrival in each village, one person would set up the EHR system and connect all devices to the LAN. A battery-operated printer (HP OfficeJet 200, Hewlett-Packard, Palo Alto, CA, USA) was available to print documents as needed.

Other than using paper health records in 2017 and EHR in 2019, there were no differences in the allocation of numbers, the information that was gathered on the health records, the workflow or the patient care process.

### Data analysis

Data obtained in 2017, the last year we used paper forms throughout the whole medical service trip, were compared to the data obtained in 2019, the first year the EHR system was exclusively utilized. The efficiency of the patient care process was defined as the number of patients served per physician per hour, which is an important determinant of the total number of patients that can be served. Each day had 2 or 3 physicians in the physician's station. The impact of EHRs on pharmacy operations was defined as the number of prescriptions that were dispensed per operational hour of the pharmacy. Data filled was either collected by hand from the paper records or exported to a Microsoft Excel file for the EHR (see Supplementary material for an example of the spreadsheet and a snapshot of the different screens of the EHR program). Statistical differences were determined by an unpaired Student's *t-*test (SPSS Statistics 27, IBM, Chicago, IL, USA) and considered statistically significant when *p* < 0.05. Graphs were created using SigmaPlot 12.3 (Systat Software Inc, San Jose, CA, USA).

## Results

### Implementation

In 2018, the electronic health record system was first implemented in the Uganda STMM after successful test runs at the School of Pharmacy. Upon arrival at each medical camp, the LAN network and EHR system were set up while the stations were set up and there was no delay in starting the medical camp while setting up the network. We encountered unanticipated technical issues on the first day and switched to paper records for day 1 and 2. After correcting the technical issues, the EHR system could be used as anticipated during the last 3 days. The tablet computers ran on their internal battery the full day and the laptop server was recharged using portable charging power packs. Based on the successful implementation in 2018, the electronic health record system was used during the 2019 medical service trip on all days and paper records were not needed at all.

### Impact of electronic health records on patient care process

**Figure 1:**
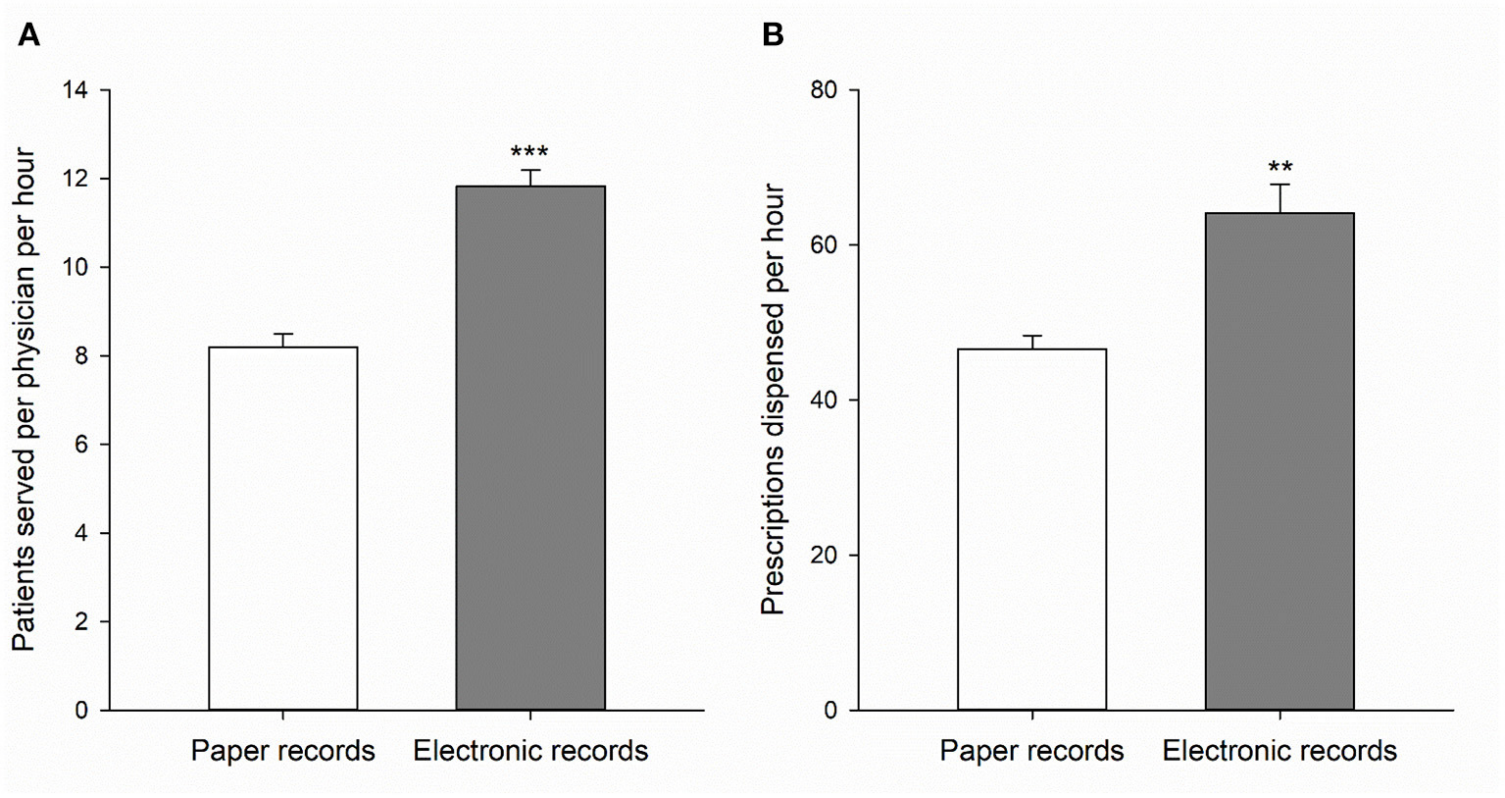
The number of patients served per physician per hour in the physician's station **(A)** and the number of prescriptions dispensed per hour in the pharmacy **(B)** when using paper health records (open bars) or electronic health records (gray bars). Data is expressed as means ± SEM of 5 individual clinic days (see Tables 2, 3). **p < 0.01 and ***p < 0.001 compared to paper records.

In the physician's station, physicians licensed to practice in Uganda performed the anamnesis and diagnosis, ordered and interpreted lab tests, and decided on the appropriate pharmacotherapy based on the Uganda clinical guidelines. The physicians were assisted by (student) pharmacists in these processes. Table 2 shows the total number of patients served per day, the total physician hours per day (where 2 physicians working 6 h each equals 12 physician hours) and the average number of patients seen per physician per hour. When using paper forms, an average of 8.2 ± 0.3 patients was served per physician per hour. An average of 11.9 ± 0.4 patients was served per physician per hour when using the EHR. Thus, 45% more patients were served per physician per hour when using the electronic records compared to paper records (*p* < 0.001, Figure 1A, Table 2).

**Table 2:** Number and flow of patients through the physician' station of the medical camp using paper or electronic health records.

**Form used**	**Day**	**Number of patients**	**Total physician hours^a^**	**Patients per physician per hour^b^**
**Paper**	1	167	19.5	8.6
	2	164	21	7.8
	3	136	15.5	8.8
	4	205	24	8.5
	5	115	16	7.2
	**Total/Mean** ^**c**^	**787**	**96**	**8.2 ±0.3**
**Electronic**	1	133	12	11.1
	2	199	16	12.4
	3	169	14.3	11.8
	4	149	13.5	11.0
	5	71	5.5	12.9
	**Total/Mean** ^**c**^	**721**	**61.3**	11.9 ±0.4***

^***^p < 0.001 compared to paper forms. ^a^Defined as the combined amount of time that the physicians were seeing patients per day, i.e. two physicians seeing patients for 5 h each equals 10 total physician hours. ^b^Total number of patients served divided by the total physician hours. ^c^Totals are shown for number of patients and total physician hours, whereas means ± SEM are shown for patients per physician per hour.

### Impact of electronic health records on pharmacy operations

In the pharmacy, the verification step was performed by licensed pharmacists only to ensure optimal patient care and the EHR allowed for quick communication with the physician's station when needed. Medication dispensing and verification in the medication use process were optimized since pertinent information could easily be read from and added to the EHR, which also served as the prescription. Table 3 shows for each day the total number of prescriptions dispensed, the total hours the pharmacy was operational, and the average number of prescriptions dispensed per hour. Although the number of patients that were served in the pharmacy per hour was not statistically different between the 2 years, 18.2 ± 1.2 (2017) and 20.7 ± 1.0 (2019) patients per hour, the average number of prescriptions per patient was 20% higher in the year we used the electronic records (2.6 ± 0.17 vs. 3.1 ± 0.10 prescriptions/patient; *p* < 0.05). As a result, 38% more prescriptions were filled per hour in the pharmacy when using electronic records compared to paper ones: 64.0 ± 3.8 and 46.6 ± 1.8, respectively (*p* < 0.01, Figure 1B, Table 3). Importantly, this higher number of dispensed prescriptions per hour in the year the electronic health record system was used was achieved with a smaller and less experienced (i.e., 5 instead of 6 licensed pharmacists and 2 instead of 5 Advanced Pharmacy Practice Experience rotation students) team from the School of Pharmacy. On average, the number of people staffing the pharmacy each day was two less in 2019 compared to 2017. In 2019, the Pharmacy also dispensed medications for patients treated by a dental team during clinic days 2, 3 and 4 with an average of 87.3 dental prescriptions per day. Since the dental team was not part of the medical camp in 2017, these dental prescriptions were excluded from the data presented in Table 3. When including these dental prescriptions, the average number of prescriptions dispensed per hour increased further to 72.0.

**Table 3:** Efficiency of pharmacy operations using paper or electronic health records.

**Form used**	**Day**	**Prescriptions dispensed**	**Pharmacy hours^a^**	**Prescriptions per hour^b^**
**Paper**	1	494	9.5	52.0
	2	420	9	46.7
	3	351	7.3	48.4
	4	412	9.5	43.4
	5	337	8	42.1
	**Total/Mean** ^**c**^	**2,014**	**43.3**	**46.6 ±1.8**
**Electronic**	1	449	7.5	59.9
	2	602	9.5	63.4
	3	550	7	78.6
	4	443	7.3	60.7
	5	201	3.5	57.4
	**Total/Mean** ^**c**^	**2,245**	**34.8**	64.0 ±3.8**

^**^p < 0.01 compared to paper forms. ^a^Defined as the amount of time from when the pharmacy was operational to start filling the first prescription until counseling of the last patient. ^b^Total number of prescriptions dispensed divided by the total pharmacy hours. ^c^Totals are shown for prescriptions dispensed and pharmacy hours, whereas means ± SEM are shown for prescriptions per hour.

## Discussion

This is the first study describing the impact of EHRs on the patient care process on a medical service trip. Although many medical service trips have been organized over the last decades (1), the impact of these have often not been studied (4). It is important to evaluate both the benefit and the unintentional harm to the people served (4, 13, 20) and to balance the needs of participating trainees and their educational programs with the benefits to the communities being served in an ethical way (9). The use of EHRs could help study the impact of medical service trips on participants and host communities by facilitating data collection and analysis and by enabling longitudinal follow up of patients in subsequent years. Various EHR systems using mobile devices have been implemented and used as reviewed by Dainton and Chu (17). Our study demonstrates that it is not only possible to implement EHRs in places where there is no access to electricity or internet, but that their use also improves the patient care process.

By switching to EHRs during medical camps in rural Uganda, we overcame challenges we faced when using paper health records, such as messiness from handling and transporting between stations, poor legibility of information entered at previous stations, and limited space to document information. Another advantage of the electronic health record system is that medical and drug information resources were installed on the tablets. Access to digital resources on a STMMs has been shown to be beneficial, particularly for participating students (22). Participants of our medical camp also had access to the local Ugandan guidelines, as advocated by Johnson et al. (10).

Importantly, we demonstrate that the use of EHRs improved the overall patient care process and flow which enabled us to serve more people per hour in their medical needs without the need to expand the team and without compromising standard of care. This is achieved by offering a more time efficient means of record keeping and communication. It was easier for physicians to refer to triage information and lab test results that had been entered into the HER, which allowed for a more efficient anamnesis and diagnosis. The EHR also allowed for more space to document clinical information and follow up of patients in the physician's station was also quicker. Moreover, data from multiple family members could easier be accessed and entered when using the EHR compared to paper forms. When using the EHR, much time was saved writing the prescriptions since the drug regimens–based on the Uganda clinical guidelines–were pre-installed and allowed for simple selection rather than handwriting them. This also improved the communication between the physicians and the pharmacy team, because there were no prescription typos or poor handwriting-related errors. Consequently, physicians spent less time clarifying information to the pharmacy team and were able to spend more time with the patients. Flow of patients between the stations could be tracked real time, which improved transitions and ensured an even flow of patients for the physicians. As a result, 45% more patients were served per physician per hour in the medical camps and 38% more prescriptions were dispensed per hour in the pharmacy when using the EHR compared to paper records. Because of this, our days in the medical camp were on average almost 1 hour shorter, which helped to not overwork the participating health care practitioners and ensured all supplies could be packed and loaded unto the bus before dark. It is important to note that the physicians did not change the standard of care when using the EHR or paper records.

The number of patients served by the physicians in our study was likely also positively impacted from the fact that the physicians were paired with a (student) pharmacist who would do the data entry into the health records, which occurred while the physician was talking to and assessing the patient. Indeed, use of medical scribes, unlicensed persons who enter information in the EHR at the direction of a health care practitioner, has been shown to improve the time efficiency and productivity of physicians (23). This benefit of a medical scribe is important as some studies reported that EHR use may increase the documentation time by physicians and nurses in regular clinical settings (24, 25), although familiarity with the system is likely to reduce documentation time and improve information flow (22). Use of EHRs has been shown to save time in other parts of the patient care process and the impact should therefore be studied at the level of the organization and patient care process as a whole rather than at the level of the single users (24). We therefore determined the impact of EHRs on pharmacy operations, the last step in the patient care process, and found that the pharmacy operated in a more time efficient manner when using EHRs–even with a lower number of people staffing the pharmacy. This is important as it ensures that the increased flow of patients from the physician's station could be processed by the pharmacy, thus improving the workflow of the entire medical camp. The impact of EHRs on pharmacy operations likely resulted from the greatly improved legibility of the patient information and prescription compared to paper records, from the fact that drug information resources were installed on the tablets which allowed for quick referencing, and from improved communication between the physician's station and pharmacy. The use of EHRs could also positively impact the overall safety of processes by preventing misinterpretation of handwriting that could potentially lead to medication errors, quick access to test results, and easy access to drug information resources. However, we did not document the number and types of pharmacist' interventions related to safety in this study using the different health records. It has previously been proposed that EHRs could improve patients' safety by improving the structure and accuracy of nursing documentation (26) and data collection (27), and by improving communication and harmonization of treatment plans (27). Since studies on the impact of EHRs on the safety of health services provided are still limited (28), future studies designed to study the impact of EHRs on safety of processes, particularly during medical camps, are needed.

Having detailed overview of the demographics, encountered disease states, and the types and number of medications dispensed in each village will optimize planning of future trips to the same or similar locations as it relates to the type and quantity of medications needed. However, post trip gathering of this data from paper forms is labor intensive as each form needs to be transcribed by hand into a spreadsheet separately and the challenges of messiness and poor handwriting remain. The ability to export data sets from the EHR system to Microsoft Excel makes data analysis easier and more time efficient. The system also has a search function to find and access the records of individual patients, which could help to follow patients longitudinally when returning to the same sites. The use of EHRs could also facilitate research studies by collecting and analyzing data, such as those related to the benefit of pharmacist interventions studies (29) and could help study the impact of STMMs on the management of chronic diseases longitudinally, which is often challenging (30). Maki et al. have developed a tool to assess the impact of STMMs on six different domains: cost, efficiency, impact, preparedness, education, and sustainability (2). Since the ability to keep records is important to evaluate these six domains (2), using EHRs will be helpful in this respect. Moreover, EHRs can serve as a tool to demonstrate standards of care were met and that the appropriate medications in the correct doses were dispensed.

There are some limitations to the study. Firstly, the teams were not identical in each year, although three of the four physicians involved participated in both years. However, the observed increased efficiency appears to be caused by the EHR rather than team composition as a similar increase in the number of patients served per physician per hour was observed in 2018, the year the EHR was used on some days and paper records on other days. Secondly, differences in clinical complexity regarding the patients could also account for differences in efficiency. However, the average number of prescriptions per patient was actually higher when the EHR was used, which may indicate a higher level of clinical complexity. Thirdly, the study is a retrospective analysis and was therefore not designed to have similar conditions between both years, although the only change that was made to the process was changing paper records to electronic ones.

Although the intent of participants of medical service trips is to do good and bring health to vulnerable populations, some have voiced concerns regarding the lack of official oversight by governmental agencies or accrediting bodies of the care provided (4, 5). This may compromise standard of care due to unnecessary or inappropriate procedures, sometimes performed by underqualified participants lacking proper supervision (5). EHR use would allow for oversight of the participants and the services provided and transfer of data to (local) governmental agencies and health care providers. Providing the patient with a printout of the health record that the patient can present to future health visits would also be beneficial to the patients and local health care providers. Permanent clinics and itinerant physicians could also benefit from the improved patient care process and increased efficiency when using EHRs.

Since familiarity with the system improves the workflow (24), we recommend training the participants on the use of the EHR system prior to the STMM under the conditions that will be present while on location, which will help to identify and correct potential technical problems in advance. Training was provided during our pre-travel preparations, which occurred in the week prior to departure. Partnering with a healthcare software company is recommended to design a system that meets the specific needs of the STMM and is user friendly, since issues with the usability of electronic health record systems are a known cause of frustration for the users (31, 32).

## Conclusions

An EHR system that does not require access to an electrical grid or the internet was successfully implemented in medical camps in rural Uganda in an interprofessional health care setting. Compared to using paper records, use of EHR leads to a more efficient workflow and patient care process where per hour more patients can be served per physician and more prescriptions can be filled in the Pharmacy. Therefore, implementation of EHRs on medical service trips is recommended to improve the efficiency of health services provided to vulnerable populations and could be a helpful tool to study the impact and quality of the health services provided.

## Data availability statement

The raw data supporting the conclusions of this article will be made available by the authors, without undue reservation.

## Author contributions

HM: conceptualization, methodology, visualization, and writing–original draft. HM and KO: funding acquisition, investigation, and data collection. HM, KO, WM, GT, MS, AN, and LR: implementation and writing–review and editing. HM, MS, AN, and LR: resources. KO: software. All authors have read and agreed to the published version of the manuscript.

## Funding

This research was funded by a Quality Initiative grant from Palm Beach Atlantic University.

## Conflict of interest

The authors declare that the research was conducted in the absence of any commercial or financial relationships that could be construed as a potential conflict of interest.

## Publisher's note

All claims expressed in this article are solely those of the authors and do not necessarily represent those of their affiliated organizations, or those of the publisher, the editors and the reviewers. Any product that may be evaluated in this article, or claim that may be made by its manufacturer, is not guaranteed or endorsed by the publisher.
